# The Change in Eating Behaviors in a Web-Based Weight Loss Program: A Longitudinal Analysis of Study Completers

**DOI:** 10.2196/jmir.3131

**Published:** 2014-11-03

**Authors:** Madeleine Svensson, Mari Hult, Marianne van der Mark, Alessandra Grotta, Josefine Jonasson, Yvonne von Hausswolff-Juhlin, Stephan Rössner, Ylva Trolle Lagerros

**Affiliations:** ^1^Halmstad UniversityDeparment of Health and Social SciencesHalmstadSweden; ^2^Karolinska InstitutetDepartment of MedicineUnit of Clinical EpidemiologyStockholmSweden; ^3^Karolinska InstitutetDepartment of Medical Epidemiology and BiostatisticsStockholmSweden; ^4^Obesity Center NorrtullStockholmSweden; ^5^Karolinska InstitutetDepartment of NeuroscienceStockholmSweden; ^6^Karolinska University HospitalDepartment of MedicineStockholmSweden; ^7^Apple Bay Obesity Research CenterStockholmSweden

**Keywords:** behavior, counseling, diet, eating, method, questionnaires, Internet, weight loss, TFEQ

## Abstract

**Background:**

Eating behaviors are essential components in weight loss programs, but limited research has explored eating behaviors in Web-based weight loss programs.

**Objectives:**

The aim was to evaluate an interactive Web-based weight loss program on eating behaviors using the 18-item Three-Factor Eating Questionnaire Revised (TFEQ-R18) which measures uncontrolled eating, emotional eating, and cognitive restrained eating. Our Web-based weight loss program is comprised of information about healthy lifestyle choices, weekly chats with experts, social networking features, databases for recipe searches, and features allowing members to self-report and track their weight, physical activity, and dietary intake on the website.

**Methods:**

On registering for the weight loss program, 23,333 members agreed to take part in the research study. The participants were then asked to complete the TFEQ-R18 questionnaire at baseline and after 3 and 6 months of participation. All data collection was conducted online, with no face-to-face contact. To study changes in TFEQ-R18 eating behaviors we restricted our study to those members who completed all 3 TFEQ-R18 questionnaires. These participants were defined as “completers” and the remaining as “noncompleters.” The relationships between sex, change in eating behaviors, and total weight loss were studied using repeated measures ANOVA and Pearson correlation coefficient.

**Results:**

In total, 22,800 individuals participated (females: 19,065/22,800, 83.62%; mean age 39.6, SD 11.4 years; BMI 29.0 kg/m^2^; males: 3735/22,800, 16.38%; mean age 43.2, SD 11.7 years; BMI 30.8 kg/m^2^). Noncompleters (n=22,180) were younger and reported a lower score of uncontrolled eating and a higher score of cognitive restrained eating. Over time, completers (n=620) decreased their uncontrolled eating score (from 56.3 to 32.0; *P*<.001) and increased their cognitive restrained eating (from 50.6 to 62.9; *P*<.001). Males decreased their emotional eating (from 57.2 to 35.9; *P*<.001), but no significant change was found among females. The baseline cognitive restrained eating score was significantly and positively associated with weight loss for completers in both men (*P*=.02) and women (*P*=.002).

**Conclusions:**

To our knowledge, this is the largest TFEQ sample that has been documented. This Web-based weight loss intervention suggests that eating behaviors (cognitive restrained eating, uncontrolled eating, and emotional eating) measured by TFEQ-R18 were significantly changed during 6 months of participation. Our findings indicate differences in eating behaviors with respect to sex, but should be interpreted with caution because attrition was high.

## Introduction

Obesity has reached epidemic proportions globally [1] and effective strategies to prevent and improve this epidemic are continuously being discussed [2]. One intervention strategy that has increased in popularity is the Web-based weight loss program, probably due to its potential for a large reach [3] in a cost- and time-effective manner [4-6]. The Internet’s potential to operate interactively makes it a valuable tool in weight loss interventions [7]. It allows for instant tailored health counseling based on the participants’ reported health behaviors, personal interests, and goals [7-9].

In 2003, our research group developed the Swedish Web-based weight loss intervention program, the Weight Club [10]. We previously studied the characteristics of members participating continuously for 6 months in the Weight Club. We found an average weight loss of 7.7% among men and 6.8% among women [11]. This is in-line with a review by Neve et al [12], who documented a minimum achieved weight loss of at least 5% in 4 of 7 Web-based studies—a weight loss considered to be sufficient to provide clinically significant health benefits [13,14].

Although the results from Web-based weight loss programs have been promising, the chance for successful weight loss may not only result from the content of the intervention or the individual’s adherence to the weight loss intervention [7,15,16], but may also be related to an individual’s lifestyle behaviors [17]. It is not unreasonable to argue that a weight loss program of any kind may influence eating behaviors, which eventually will lead to weight loss. Conversely, some may argue that a change in eating behavior may be the primary mechanism that results in a loss of weight, independent of intervention.

However, only a few studies have examined the association between individuals’ eating behaviors and weight loss or weight gain, with controversial results. Results from some studies suggest a relationship between high levels of restrained eating behavior, defined as an individual’s conscious food restriction to control body weight [18], and lower body mass index (BMI) [19-22]. Although, in a review by Lowe et al [23], normal-weight individuals with high levels of restrained eating behavior or with a history of dieting seemed to be more susceptible to gain weight compared with those with low restrained eating or no history of dieting. Alternatively, other researchers found no associations between restrained eating and BMI [24].

Moreover, the tendency to lose control over eating when feeling hungry or being exposed to external stimuli [25,26] has been shown to correlate with an individual’s overeating and impulsive eating, contributing to increased bodyweight [27,28]. This eating behavior has previously been referred to as “disinhibited eating” [18], but has been revised to the term “uncontrolled eating behavior” [26]. It has been suggested that emotional eating, overeating in response to negative emotions or stimuli, is a learned response rather than a consequence or a mediator for overweight or obesity [29-31]. Koenders et al [32] stress that it is “emotional eating, rather than lifestyle behaviors, [that] drives weight gain” among people with overweight and obesity.

The possible change of eating behavior over time has not been fully investigated. Even less is known about how eating behavior may change over time while partaking in a Web-based weight loss program and if there is a relationship between eating styles and weight loss. To disentangle these matters, this study was designed to examine eating behaviors in a large cohort of members participating in the Swedish Web-based Weight Club for 6 months. We specifically studied the participants’ eating behaviors over time, using the 18-item Three-Factor Eating Questionnaire Revised (TFEQ-R18).

## Methods

### Weight Loss Program

**Figure 1:**
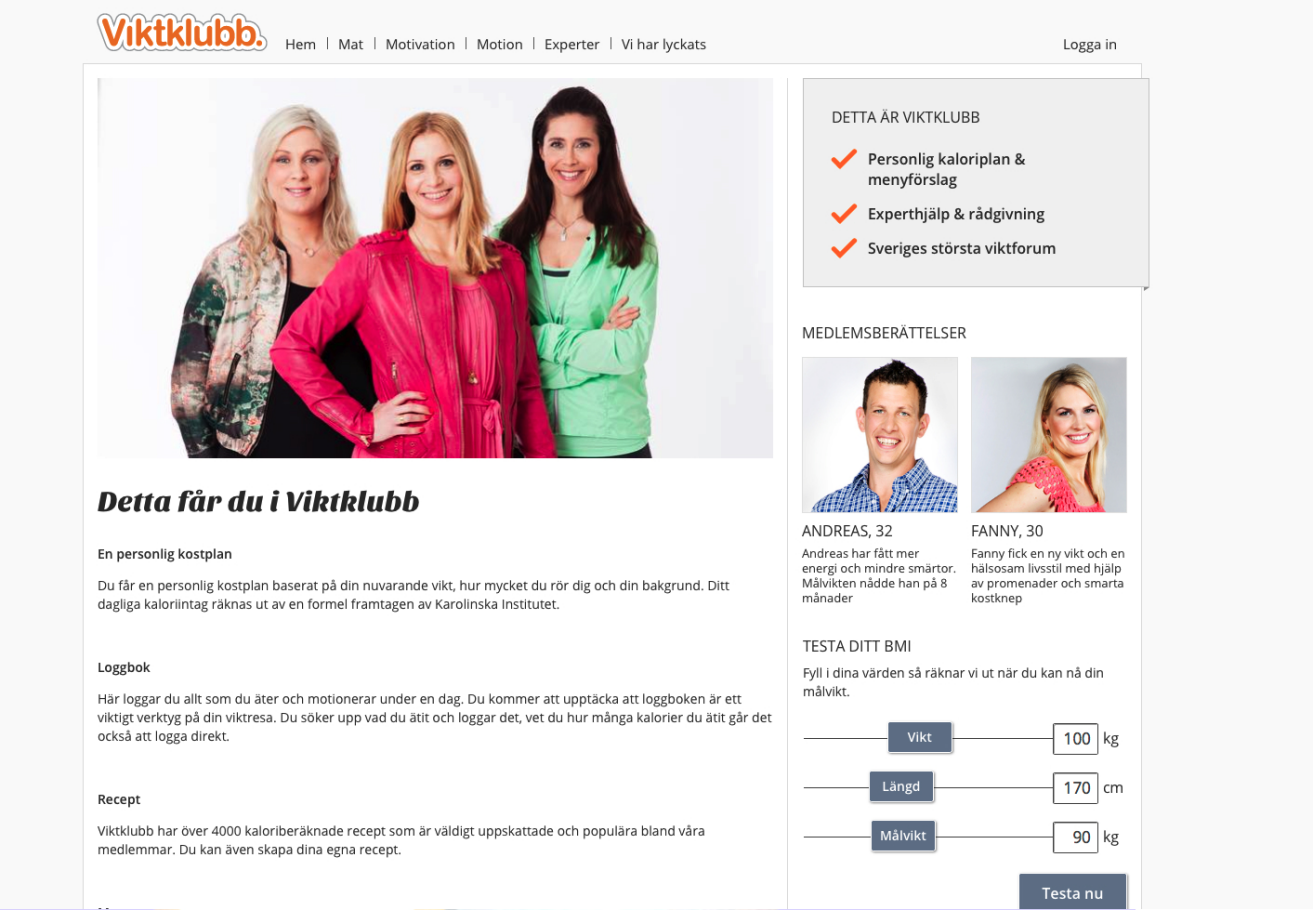
Home page for Viktklubb (Weight Club) with information about what is included in a membership, success stories, BMI calculator, and setting current and future goals.

The participants were registered members of a Swedish Web-based weight loss program [10], who were invited through a media advertising campaign. The program was specifically tailored to the general Swedish population. The weight loss club was named the Weight Club (Swedish: Viktklubb) and was developed in collaboration with health professionals (physicians, dietitians, nurses, and researchers) at the Karolinska Obesity Unit, Karolinska University Hospital Stockholm, Sweden, and the Swedish newspaper *Aftonbladet* [33] (see Figure 1).

The Weight Club [10] was accessible on a 24-hour basis. At the start, members were asked to weigh themselves and to record and report their weight once a week. The recommended weight loss was ≤1 kg per week. Recommendations for daily energy intake were calculated using the Benedict formula [34]. Approximately 1000 meals and recipes by well-known Swedish chefs were accessible and regularly updated. All meals were based on guidelines from the national Swedish Food Agency. The participants had the opportunity to modify or create their own recipes and use the search feature to evaluate their food choices regarding nutritional content.

**Figure 2:**
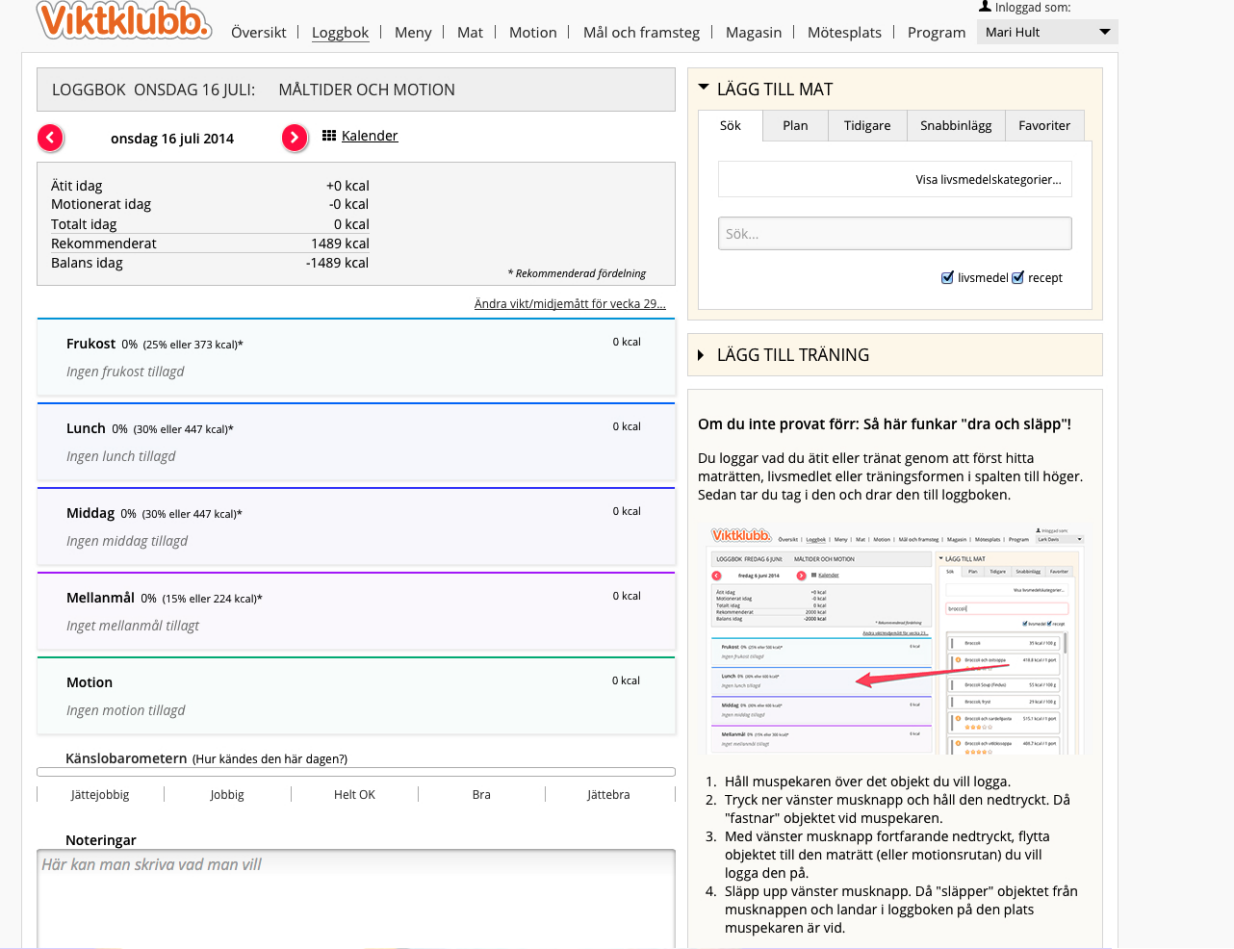
A diary for daily food intake, level of physical activity, and additional private comments about the day (using a drag-and-drop function). The balance between actual energy intake minus energy expended through physical activity compared to suggested energy intake is seen in the upper left corner.

Members were instructed to frequently record their weight, food intake, and physical activity level using the online food and exercise diaries (see Figure 2). The members automatically received feedback. This feedback was communicated to the members through the website’s interactive charts and figures presenting their progress (ie, with respect to weight loss and frequency of physical activity). Emails with tips and advice on how to change eating behaviors to encourage weight loss and weight maintenance were sent on a regular basis. In addition, chats were available on the website allowing for exchange of knowledge, experiences, and social support during the weight loss process. The members could also use a personal blog—a feature used by 25% of the members. Additionally, members had the opportunity to participate in weekly online chats with a physician or dietician for further personal advice and support. Questions and answers from the chats were published on the newspaper’s website and at the Weight Club’s website on a weekly basis. Members who had successfully managed to lose weight were interviewed by the Weight Club team and these interviews were posted online.

### Participants and Design

To access the weight loss program, the members had to subscribe to a 3-, 6-, or 12-month membership plan (prices ranging from €33 to €55) [10]. All members were asked to answer a questionnaire of sociodemographics and whether they were interested in participating in the research study. The consenting participants were then asked to answer questions about age, weight, and height and fill out the TFEQ-R18 at baseline, 3 months, and 6 months.

The TFEQ is a widely used validated questionnaire to measure eating behaviors among heterogeneous populations [26,30,35-37]. The TFEQ-R18 is an 18-item revised version [26] of the original questionnaire, developed by Stunkard and Messick, which was 51 items originally [18]. The TFEQ-R18 encompasses 3 concepts of eating behaviors including cognitive restrained eating (6 items), emotional eating (3 items), and uncontrolled eating (9 items) [26,37,38]. The TFEQ-R18 is based on scores, wherein each item has a score. The total scores are then summed and the results are presented on a scale of 0-100, where higher values indicate a greater degree of that particular behavior [39].

A total of 23,233 members agreed to participate in the study. Of these, 22,844 members submitted complete information on sex, age, weight, and the TFEQ-R18 at baseline. Overall, 37 participants were excluded from the data analysis due to obviously conflicting answers (ie, unrealistic BMI and weight goal). Also, to prevent confounding due to bariatric surgery, cancer, or other reasons with the potential to alter eating behaviors, we omitted 7 participants because they reported a weight loss of more than 30% in 6 months.

To study members’ eating behaviors (cognitive restrained eating, uncontrolled eating, and emotional eating) over time, we restricted our analyses to members who were participating continuously for 6 months because this was a typical time to sign up for the weight loss program. Because the Weight Club was open to the public (and not limited to solely study participants or patients), members entered and left the program on a voluntary basis. Hence, we only have data on those members who agreed to take part in the study and who submitted the research questionnaires.

We defined 6-month compliance by restricting our analyses to participants who registered their weight at least once during the past month and logged on at least twice during the first 3 months and twice during the second 3 months of participation. As a result, 4426 participants were eligible for study.

**Figure 3:**
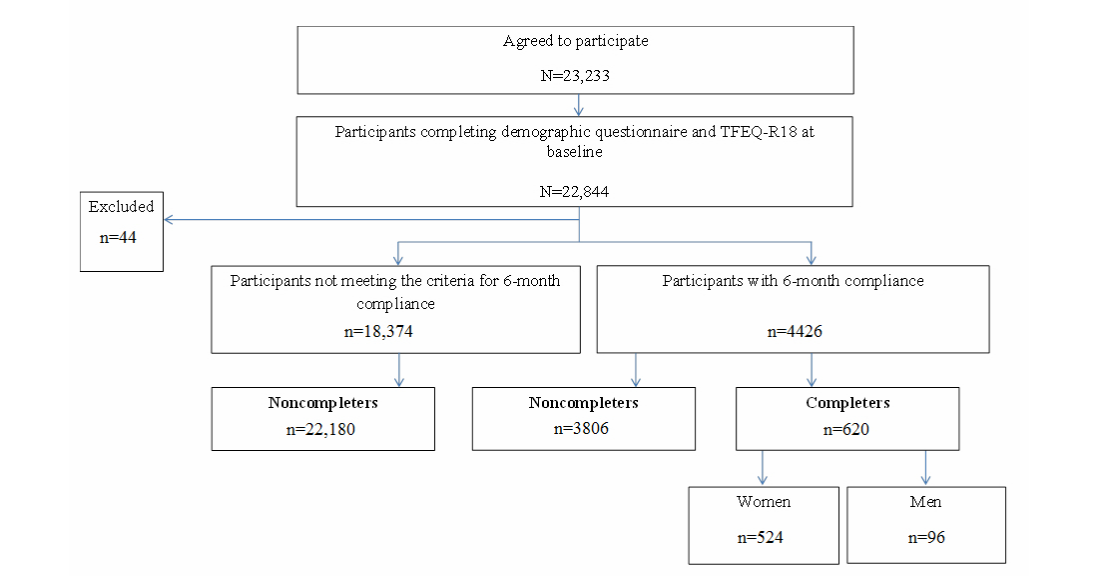
Flowchart of the study design.

To study changes in eating behaviors over time, we further restricted our analyses to those participants who had completed the baseline questionnaire and the TFEQ-R18 at baseline and at 3 and 6 months, leaving 620 participants from our primary study sample. Those participants who met these 2 criteria (1) 6-months compliance and (2) submitting complete data (baseline questionnaire and TFEQ-R18 at baseline and at 3 and 6 months) were categorized as “completers.” Those participants not meeting these criteria were categorized as “noncompleters.” See Figure 3 for a flowchart of the study design.

All data were collected through the website’s database and sent to the researchers on a regular basis. The Ethics Committee of the Karolinska Institutet approved the study.

### Statistics

Descriptive statistics (mean, SD) were computed to summarize the participants’ baseline characteristics (ie, age, BMI, TFEQ-R18 scoring, and years of education). Results were stratified by sex and by completers and noncompleters. The Mann-Whitney *U* test was computed to study possible differences between completers and noncompleters for continuous variables, such as age, BMI, and TFEQ-R18 scoring. The Pearson chi-square test was computed to test for differences in categorical variables (level of education) between completers and noncompleters.

The reported eating behaviors and weight level were summarized at baseline and at 3 and 6 months. We also summarized the total number of log-ins to the website during their 6-month participation.

We studied the participants change in eating behavior over time and potential differences between sexes using a repeated measures analysis of variance (ANOVA). In these analyses, the measured eating behavior at 3 time-points was analyzed as a within-subjects factor, whereas the participants’ sex was analyzed as a between-subjects factor. The interaction between sex and time was also tested. If significant, repeated measures ANOVA was carried out separately for men and women.

Pearson correlation coefficient (*r*) was used to study the relationship between change in eating behaviors over 6 months and total weight loss percentage, computed as ([weight at the beginning–weight after 6 months]/weight at the beginning)*100. We also studied the relationship between change in eating behaviors over 6 months and the total number of log-ins to the website using the same analysis.

*P* values less than .05 were considered statistically significant. All analyses were performed using SPSS 15.0 for Windows (SPSS Inc, Chicago, IL, USA).

## Results

At baseline, the mean age of the 22,800 study participants was 39.6 (SD 11.4) years for females and 43.2 (SD 11.7) years for males. More than 80% (19,065/22,800, 83.62%) of the participants were females. The mean BMI was 29.0 (SD 5.0) kg/m^2^ for females and 30.8 (SD 4.3) kg/m^2^ for males.

When studying differences in baseline data between completers and noncompleters, we found that both male and female completers were slightly older (*P*<.001 and *P*=.002, respectively) compared to noncompleters. Female completers also had a higher BMI (*P*<.001), and were more educated (*P*=.02) compared to noncompleters, whereas no significant difference was found in BMI or education between male completers and noncompleters. (Table 1). The average weight loss for male completers was 7.0% (SD 5.1) and 5.8% (SD 5.0) for female completers.

**Table 1:** Baseline characteristics of the study participants (N=22,800).

Characteristics		Women				Men			
		All n=19,065	Completers n=524	Noncompleters n=18,541	*P* ^a^	All n=3735	Completers n=96	Noncompleters n=3639	*P* ^a^
Age (years), mean (SD)		39.6 (11.4)	43.4 (11.8)	39.5 (11.3)	<.001	43.2 (11.7)	46.6 (10.5)	43.1 (11.7)	.002
BMI (kg/m^2^), mean (SD)		29.0 (5.0)	29.8 (4.9)	29.0 (5.0)	<.001	30.8 (4.3)	31.5 (4.3)	30.7 (4.3)	.07
**Eating behavior, mean (SD)**									
	Uncontrolled eating	51.8 (15.1)	56.3 (14.4)	51.6 (15.1)	<.001	51.7 (14.8)	56.8 (13.6)	51.5 (14.8)	.001
	Emotional eating	43.5 (30.3)	45.1 (28.6)	43.5 (30.4)	.14	55.3 (28.9)	57.2 (28.4)	55.3 (28.9)	.57
	Cognitive restrained eating	48.6 (11.4)	50.6 (10.7)	48.5 (11.4)	.002	47.2 (12.2)	50.8 (10.9)	47.1 (12.2)	.09
**Education, n (%)**					.02				.56
	≤9 years	1253 (6.6)	24 (4.5)	1229 (6.6)		312 (8.3)	6 (6.3)	306 (8.4)	
	10-12 years	9176 (48.1)	235 (44.8)	8941 (48.2)		1840 (49.3)	45 (46.8)	1795 (49.3)	
	≥13 years	8097 (42.5)	244 (46.6)	7853 (42.4)		1484 (39.7)	44 (45.8)	1440 (39.6)	
	Unknown	539 (2.8)	21 (4.0)	518 (2.8)		99 (2.7)	1 (1.0)	98 (2.7)	

^a^ *P* value based on Mann-Whitney *U* test (age, BMI, uncontrolled, emotional, and cognitive restrained eating behavior) or Chi-square test (education).

Both male and female completers reported higher baseline scores of uncontrolled eating compared to noncompleters (men: *P*=.001; women: *P*<.001), although no significant difference in baseline emotional eating score was found. Female completers reported higher baseline scores of cognitive restrained eating (*P*=.002) compared to female noncompleters, whereas no significant difference was observed among men (Table 1).

**Figure 4:**
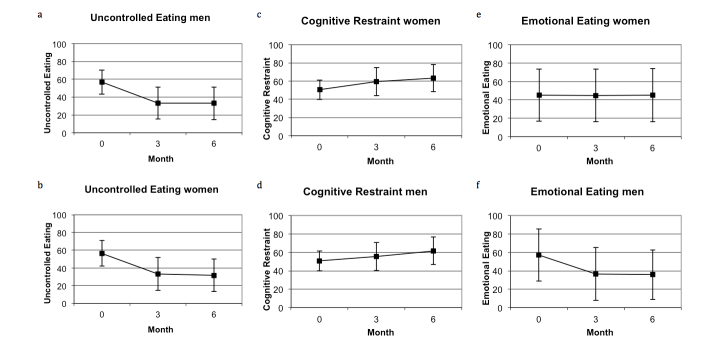
Change over time in uncontrolled eating behavior a) among women (n=524) and b) among men (n=96). Change over time in cognitive restraint eating behavior c) among women (n=524) and d) among men (n=96). Change over time in emotional eating behavior e) among women (n=524) and f) among men (n=96).

The results from repeated measures ANOVA (Table 2) suggest no interaction between time and sex for uncontrolled eating behavior (*P*=.76). The variable time was significant (*P*<.001), and both male and female completers significantly decreased their uncontrolled eating score over time. For cognitive restrained eating behavior, there was no significant interaction between time and sex (*P*=.12), but the variable time was significant (*P*<.001) with both males and females increasing their cognitive retrained eating over time (see Figure 4a-d).

An interaction between time and sex for emotional eating (*P*<.001) was observed (Table 2). The effect of time was significant in men (*P*<.001), but not in women (*P*=.98). Males decreased their emotional eating score from 57.2 at baseline (SD 28.4) to 35.9 (SD 26.9) after 6 months participation. Females, conversely, had a constant emotional eating score (Figure 4e,f). Thus, our results suggest a difference in change of emotional eating behaviors over 6 months with respect to sex.

**Table 2:** Results of repeated measures ANOVA.

Eating behavior		*F* ratio (*df*)^a^	*P* value
**Uncontrolled eating behavior**			
	Sex	0.30 (1, 618)	.58
	Time	193.93 (1.17, 720.22)	<.001
	Interaction (time×sex)	0.13 (1.17, 720.22)	.76
**Cognitive restrained eating behavior**			
	Sex	2.72 (1, 618)	.10
	Time	73.43 (1.64, 1016.24)	<.001
	Interaction (time×sex)	2.24 (1.64, 1016.24)	.12
**Emotional eating behavior**			
	Sex	1.58 (1, 618)	.21
	Time	12.21 (1.16, 717.50)	<.001
	Interaction (time×sex)	11.77 (1.16, 717.50)	<.001

^a^ Degrees of freedom for time and interaction are adjusted according to Greenhouse-Geisser correction for nonsphericity.

Our results suggest an association between change in eating behaviors and total weight loss among our completers. Weight loss was negatively correlated to changes in scores for emotional eating (women: *r*=–.12, *P*=.01; men: *r*=–.24, *P*=.02), suggesting that reductions in emotional eating are associated with reductions in weight. Analogously, weight loss was negatively correlated to changes in scores for uncontrolled eating, although not significant for men (women: *r*=–.11, *P*=.02; men: *r*=–.19, *P*=.07), suggesting a possible association between reductions in uncontrolled eating scores and reductions in weight. Cognitive restrained eating scores increased during program participation and changes in the score were positively correlated to weight loss among our females (*r*=.11, *P*=.01), but not among males (*r*=.03, *P*=.80). Thus, results suggest a possible association between reductions in weight and an increase in cognitive restrained eating score over 6 months in females.

We found no significant correlation for female or male completers between their total number of log-ins to the website and change in eating behaviors. The correlations were low between total log-ins vs change in emotional eating (women: *r*=–.02, *P*=.63; men: *r*=–.08, *P*=.44), uncontrolled eating (women: *r*=–.01, *P*=.85; men: *r*=–.09, *P*=.36), and cognitive restrained eating (women: *r*=–.01, *P*=.80; men: *r*=.01, *P*=.89).

## Discussion

In this Web-based weight loss program, eating behavior changed during the 6-month program participation. We found that male participants changed all eating behaviors; cognitive restrained eating scores increased over 6 months, whereas uncontrolled and emotional eating scores decreased. Among our female participants, cognitive restrained eating increased and uncontrolled eating decreased during the program.

The effect of sex with respect to emotional eating is controversial in existing research. In our cohort, there was no significant difference between male and female participants’ emotional eating scores at baseline. We cannot exclude that an underlying selection process leading people to join the program may have affected baseline values, making them partially different from other cohorts in which different enrollment criteria were used. Interestingly, Ozier et al [40] found that men reporting high levels of emotional eating were almost 3 times as likely as women to be overweight. Because our participants were overweight at baseline, we were not able to make this comparison with respect to BMI and sex. However, Konttinen et al [41] reported greater tendencies among women to report higher emotional eating scores than men, a result also supported by Karlsson et al [26] in the Swedish Obese Subjects (SOS) study. In their study, half of all participants (n=4377) scored at the top of the TFEQ emotional eating scale. Almost 40% of male participants and 60% of female participants reported high emotional eating [26]. Hence, prior literature is in disagreement regarding the effect of sex on emotional eating.

Interestingly, Péneau et al [42] reported that former or current dieters had higher scores of emotional eating compared to those who had never dieted. A possible explanation for this change is the tendency to restrict food intake during specific time periods with a high number of relapses, which may contribute to increased risk for overeating caused by emotional cues [42,43]. Presumably, the participants who took part in our Web-based weight loss intervention have had a similar history of dieting as those described previously. It is likely that our participants, before signing up to the Weight Club, characterized the behaviors of a dieter, which in turn may have contributed to the amplified emotional eating score at baseline. Our male participants scored 57.2 and our female participants scored 45.1 on the 100-point TFEQ scale. These scores are in-line with the scores of an obese Swedish sample (BMI 44.5 kg/m^2^) scoring eating behavior before gastric bypass with TFEQ [44]. Interestingly, in a community-based French cohort with leaner subjects who might have a different history of dieting (the average BMI among the male participant was 26 kg/m^2^ and 25 kg/m^2^ among the female participants) the scores for emotional eating were much lower in men (TFEQ score=22), whereas women scored only slightly lower (TFEQ score=43) [37]. This may suggest that the background history of dieting should be considered in future weight loss interventions, particularly in men because it might affect the overall eating behavior and require personalized treatment methods to optimize weight loss.

Furthermore, the correlation between increased cognitive restrained eating behavior and weight loss for females that we found was put forward by Stunkard and Messick 30 years ago [18]. Other researchers proposed lowered BMI as a result of cognitive restrained eating behaviors [19-21]. Cognitive restrained eating has, for example, been reported to be associated not only with low energy intake in a randomized weight loss intervention study [45], but also with long-term weight loss [46]. An increased cognitive restrained eating behavior may generate an overall improved self-control over food intake [47,48]. However, it should be emphasized that our results on eating behaviors do not imply causality, although it is conceivable that behaviors related to cognitive restrained, uncontrolled, and emotional eating may have an impact on food intake and ultimately weight loss.

The present study comprised a baseline sample of 22,844 participants submitting data on baseline characteristics and TFEQ-R18. To our knowledge, this is the largest Web-based research study conducted on eating behaviors to date, even if our study demonstrates a high prevalence of participant attrition. Neve et al [49] reported that commercial Web-based weight loss programs generally show high attrition rates. The authors propose a relationship between higher nonusage attrition and age, exercise level, emotional eating habits, eating breakfast, and skipping meals. A careful drop-out analysis investigating when the participants left the study with respect to basic characteristics, health aspects, and TFEQ-scores would have strengthened our study. However, such an analysis is difficult to conduct in a commercial Web-based weight loss program due to our inability to control compliance.

The absence of a control group prevents us from concluding that the observed changes in eating behaviors actually occurred as a result of participating in our Web-based weight loss program. Besides, we did not see a statistical relationship between the frequency of log-ins and greater change in eating behavior. We cannot exclude the possibility that any dieting person would experience similar changes over a 6-month period. Or indeed, that the simple act of repeatedly answering questions about eating behavior may cause one to adjust one’s eating behavior or cause an increased awareness of one’s eating behavior, which would affect subsequent self-reports [50]. For example, recent evidence suggests that self-reports of emotional eating reflect concern over emotional eating, rather than the actual act of eating when feeling emotional [22,51].

Our final analyses included a total of 620 participants, of which 96 were men. It is not surprising that more than 80% of our baseline participants were women; rather, this is a common phenomenon in health research [52]. Additionally, the completers in our study were older than the noncompleters, supporting previous research on age groups engaged in Web-based weight loss programs, in which older participants have shown higher compliance rates than younger ones [15,53,54].

According to recent statistics, Swedish citizens older than age 45 years are less likely to use the Internet on a daily basis compared to younger citizens, primarily due to lack of interest [55]. However, according to a report on Internet statistics, almost 90% of the Swedish population had access to the Internet in 2012 [55] suggesting high computer literacy in the Swedish population. Yet Web-based health interventions require certain levels of health technology literacy among the participants—the skills to read, understand, and personalize health information communicated via the Web and to be able to transform this information into action [56]. A selection of participants with high level of health technology literacy is thus possible in the present study. Therefore, older individuals who have access to the Internet may be an important target group in Web-based interventions because they seem more receptive to this type of intervention.

Although this study admits its limitations, our results add further understanding of baseline eating behaviors among overweight individuals and how eating behaviors changed during 6 months participating in a Web-based weight loss program. Enhanced knowledge about eating behaviors among individuals taking part in weight loss intervention programs might open opportunities for health professionals to personalize treatment and overall health care, meeting the needs and preferences of the target group.

This Web-based weight loss intervention suggests that eating behaviors (cognitive restrained eating, uncontrolled eating, and emotional eating) measured by TFEQ-R18 were significantly changed during 6 months of participation. Cognitive restrained eating scores increased and uncontrolled eating scores decreased among both male and female participants, whereas emotional eating scores only decreased among male participants. Our findings indicate differences in eating behaviors with respect to sex, but should be interpreted with caution because attrition was high.

## Abbreviations

BMI: body mass index
SOS study: Swedish Obese Subjects study
TFEQ: Three-Factor Eating Questionnaire
TFEQ-R18: 18-item Three-Factor Eating Questionnaire Revised
